# Ancient DNA, lipid biomarkers and palaeoecological evidence reveals construction and life on early medieval lake settlements

**DOI:** 10.1038/s41598-021-91057-x

**Published:** 2021-06-03

**Authors:** A. G. Brown, M. Van Hardenbroek, T. Fonville, K. Davies, H. Mackay, E. Murray, K. Head, P. Barratt, F. McCormick, G. F. Ficetola, L. Gielly, A. C. G. Henderson, A. Crone, G. Cavers, P. G. Langdon, N. J. Whitehouse, D. Pirrie, I. G. Alsos

**Affiliations:** 1 Tromsø Museum, Artic University of Norway, Tromsø, Norway; 2 School of Geography and Environmental Science, University of Southampton, Southampton, UK; 3 School of Geography, Politics and Sociology, Newcastle University, Newcastle upon Tyne, UK; 4 School of Geography, Earth and Environmental Sciences, University of Plymouth, Plymouth, UK; 5 IMSET, Bournemouth University, Poole, UK; 6 Department of Geography, Durham University, Durham, UK; 7 Archaeology, Queens University, Belfast, Northern Ireland UK; 8 Department of Environmental Science and Policy, University of Milan, Milan, Italy; 9 LECA, Laboratoire d’Ecologie Alpine, Université Grenoble Alpes, Université Savoie Mont Blanc, CNRS, Grenoble, France; 10 LECA, Université Grenoble Alpes, Université Savoie Mont Blanc, CNRS, Grenoble, France; 11 AOC Group Ltd., Edinburgh, Scotland UK; 12 Department of Archaeology, School of Humanities, University of Glasgow, Glasgow, UK; 13 School of Applied Sciences, University of South Wales, Pontypridd, UK

**Keywords:** Biological techniques, Ecology, Ecology, Environmental social sciences, Limnology

## Abstract

Direct evidence of ancient human occupation is typically established through archaeological excavation. Excavations are costly and destructive, and practically impossible in some lake and wetland environments. We present here an alternative approach, providing direct evidence from lake sediments using DNA metabarcoding, steroid lipid biomarkers (bile acids) and from traditional environmental analyses. Applied to an early Medieval Celtic settlement in Ireland (a crannog) this approach provides a site chronology and direct evidence of human occupation, crops, animal farming and on-site slaughtering. This is the first independently-dated, continuous molecular archive of human activity from an archeological site, demonstrating a link between animal husbandry, food resources, island use. These sites are under threat but are impossible to preserve in-situ so this approach can be used, with or without excavation, to produce a robust and full site chronology and provide direct evidence of occupation, the use of plants and animals, and activities such as butchery.

## Introduction

Globally, societies have created settlement structures in lakes and wetlands—for a variety of reasons that are poorly understood^1,2^. Examples include wetland mounds in SE and central America, Neolithic lake villages in Europe, and wetland islands from Scandinavia to Australia^1^. If we can determine what, and when, activities took place on these sites, then they can inform us about the economic, cultural and environmental drivers impacting societies that invested in such enigmatic and expensive structures. However, these sites are difficult and expensive to excavate, being surrounded by water or high water-tables. Also, as with dryland excavation, cultural material is either damaged or requires expensive and difficult post-excavation conservation.

**Figure 1:**
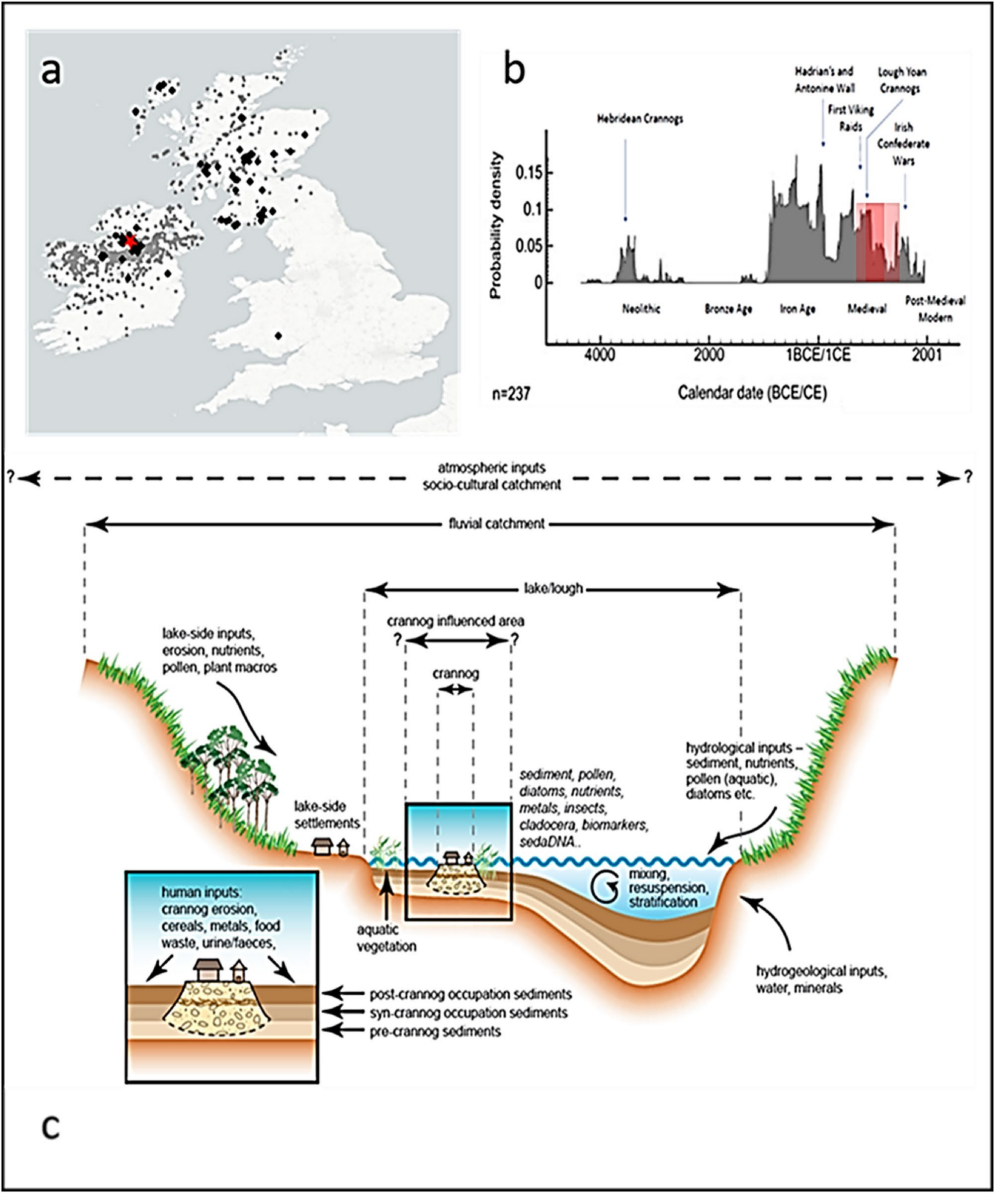
**(a)** The distribution of all crannogs in Celtic Britain and Ireland (grey circles), and crannogs with radiocarbon dates (black diamonds) collated as part of this study. The red star indicates the location of the study site, **(b)** age distribution of all dated crannogs based on the summed probability distribution of calibrated ^14^C dates, with the red box covering the period of activity at Lough Yoan, and **(c)** typical environmental context and catchment areas for different proxies around a crannog-like lake settlements.

We test here the hypothesis that the construction of, and life on an archaeological site in a lake can be detected and identified from both ancient DNA and lipid biomarkers from the adjacent lake sediments and in doing so present an innovative molecular approach to studying ancient human settlements. The type of wetland site used here, a crannog, is an entirely, or largely, artificial island in a lake or lough/loch (freshwater or marine) or in a wetland system—typical of the Atlantic Seaboard and Celtic fringe (from Scotland to NW France). In Ireland there are an estimated 1200 crannogs of which only 28 have had any excavation and mostly these have been old excavations from drained lakes^2^. In Scotland there are an estimated 600 crannogs^3^, of which only 7 have been excavated since 1950 CE with most of these being from drained wetlands due to the difficulty and high cost of excavating small water-surrounded sites (Fig. 1a ^2^). Outside this area crannogs are rare, but include singe examples from Wales (Llangorse) and Denmark (Solvig)^4,5^, although lake and wetland villages are particularly common across Europe^1^. Due to the high water-table and subsidence, these sites can have spectacular preservation of artifacts, especially organic materials such as wood and textiles^4^. Unfortunately, many sites are under threat due to the slow natural decay of associated timbers, subsidence, and erosion as a result of forest clearance on sites, changes in lake level and recreational activities^6^—as has been demonstrated by underwater surveys^7^. There is both an archaeological and heritage need to understand more about the construction, use and abandonment of these remarkable sites before they are lost due to both natural and human-induced erosion, especially since preservation in situ is practically impossible.

Crannogs were constructed predominantly during the European Iron Age (c. 800–1 BCE) through to the early Medieval period (400–1300 CE; Fig. 1b), in both Ireland and Scotland^3^, although isolated examples occur outside these time-windows from as early as the European Neolithic^8^ and as late as the post-Medieval period (1300–1700 CE), especially in Ireland^9,10^. The functions and settlement status of crannogs have been debated for over a hundred years^11^—were they permanently settled fortifications, residential houses or storehouses, or a combination of these structures. Archaeological and historical evidence for a range of activities (crop storage and/or processing, animal pens, tanning, brewing, weaving, metalworking) is available but this is only ascertainable with full excavation. Traditional archaeological approaches have emphasized a wide variety of sociocultural factors including the needs of the elite (as palaces), as safe-places for people and valuable property (as protective stores) as well as cultural and ritual associations with water, myth/legends, religion and the display of wealth and power, which is certainly attested for some of the later examples^12,13^.

Our study takes advantage of the lacustrine site setting in order to apply a novel combination of existing and new palaeolimnological techniques to cores at the site margin. After a stratigraphic survey, a location is chosen, which is still through the sedimentary material stratigraphically associated with the island, but far enough out to not damage any of the site structure (such as revetments) and outside the scheduled area which is protected by law. In practice this is normally 10–15 m out from the outer wooden piles towards the lake center. This non-invasive archaeological investigation avoids any damage to the structures and acts as an exemplar of what might be achievable for a much larger range of wetland sites including islands (as here), villages and pile-dwellings worldwide. We apply here a novel combination of molecular methods (ancient sediment DNA—sedaDNA), lipid biomarkers, elemental scanning systems using Quantitative Evaluation of Materials Scanning Electron Microscopy (QEMSCAN), X-Ray Fluorescence (XRF) and traditional palaeolimnological methods (pollen, diatoms, chironomids) to sediments collected adjacent to two crannogs in a small lake in Northern Ireland (Supplementary Information Fig. 1). The aim is to provide a proof of concept for the detection of human activity from a lake-dwelling site without conventional archaeological excavation, although augmented by one through-crannog core and some historical finds (Supplementary Information Table 1). This approach utilises the ‘biogeochemical halo’ around such sites that occurs at lake edges in small, shallow and protected lakes in which full water-mixing is limited (Fig. 1c, ^14,15^). The approach is based on the proposition that most human activities produce a direct biogeochemical trace (particularly DNA, bile acids and bone) in adjacent lake sediments, although this will be dependent on lake conditions and nature of these activities. With this approach we demonstrate a remarkable record of site construction, use and abandonment. The techniques used were chosen deliberately to cross-validate interpretations of independent lines of evidence, such as mammal sedaDNA and bile acids. The ability to identify, and date, specific human activities without excavation of wetland and lake archaeological sites from adjacent sediment cores could revolutionise wetland archaeology.

## Results

### Providing a site chronology

**Figure 2:**
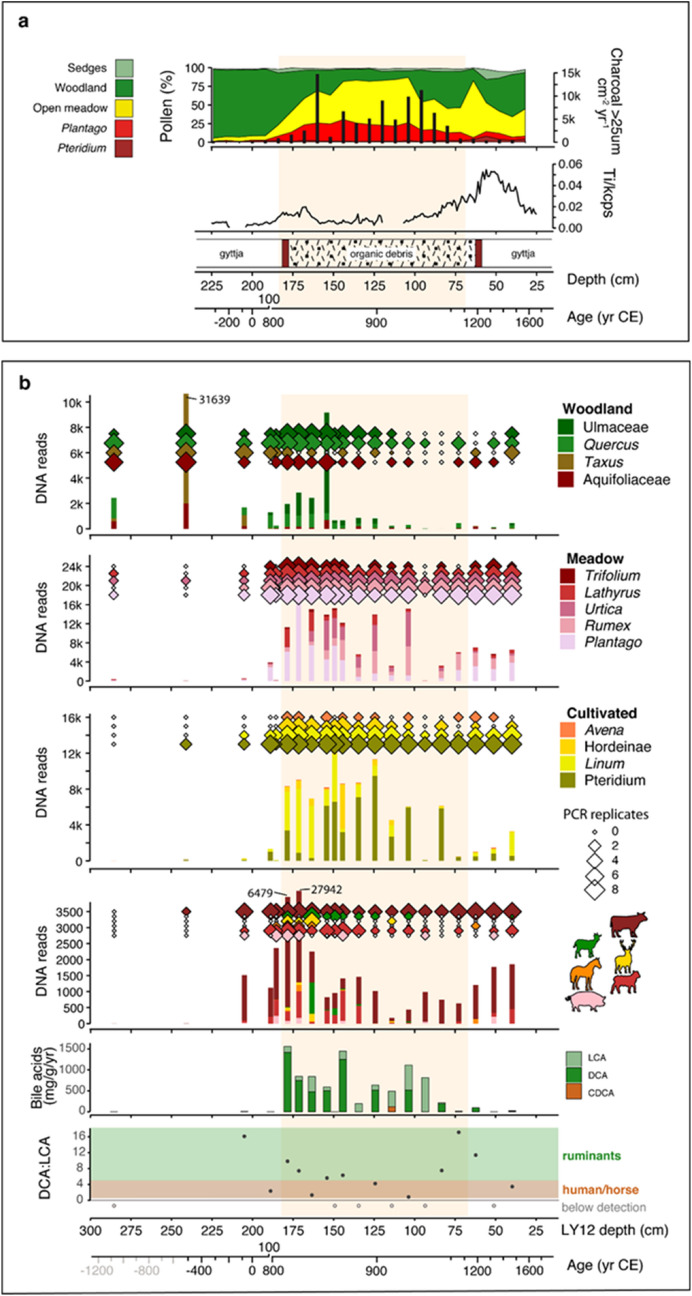
High-resolution analysis of terrestrial indicators in sediment cores taken near the northern crannog in Lough Yoan. **(a)** Pollen, charcoal fluxes, and stratigraphy. **(b)** Sedimentary DNA (*seda*DNA) from woodland, meadow and cultivated plants, domesticated mammals and ratios of bile acids specific to different animal groups.

Age depth modelling using AMS radiocarbon dates along with XRF core scanning, particularly of titanium (Ti) as an indicator of the addition and erosion of minerogenic material, has provided a chronology for the site. This is challenging as coring sequences adjacent to and through sites are non-linear. We use the age-depth model that fits best to Ti (Fig. 2a; Supplementary Information Fig. 2; Supplementary Information Table 2), and the stratigraphic changes in wood and charcoal to construct individual site chronologies (Supplementary Information data Table 3). Using this method the construction of the northern site with a basal timber has a modelled age of 827 ± 44 CE (95% CI), which also coincides with a reduction of woodland (pollen) and increased charcoal from 188 cm depth around the northern site. The start of high Ti values indicating erosion in this case suggests a reduction in intensity of use at 936 ± 31 CE with final abandonment probably at 1203 ± 29 CE. In contrast using the best model for the southern site we have the initial opening of the landscape at 61 ± 41 BCE and first charcoal in pollen slides at 236 ± 76 CE and construction with the first basal timber at 666 ± 41 CE. Initial site erosion is dated to 921 ± 33 CE and the end of the first period of use with the top timber is dated to 986 ± 47 CE. A later Medieval re-occupation is dated to 1490 ± 25 CE ending around 1527 ± 26 CE. Although the southern site was constructed earlier than the northern site (seventh century vs ninth century), both sites go out of use at the same time in the tenth century. The southern site is then re-occupied around 500 years later. Given the overlap in their construction and use in the 9^th^ to early tenth centuries and the small size of the lake it is highly likely that they belonged to the same social group and local chief, or elite. After construction the sites seem to be used for ca. 255 (range 183–327 for southern crannog) and 110 years (range 34–184 years for the northern crannog) in a period of still widespread, but declining crannog construction (Fig. 1b).

### SedaDNA metabarcoding

The results from pollen (Fig. 2a) and sedaDNA from the northern site (Fig. 2b) show that the crannog was constructed in a forested landscape. Crannog establishment was associated with a period of woodland clearance (starting c. 827 CE), leading to an almost totally cleared local landscape by the end of the ninth century CE. The period of crannog use corresponds to an increase in taxa associated with open ground and agricultural activity—particularly grazing—and high read numbers and PCR replicates of bracken (*Pteridium*) (Fig. 2b). This is accompanied by the appearance of cultivars of cereals (barley and oats) and flax (Fig. 2b). The mammal sedaDNA showed major changes at the start of the crannog use in cattle (*Bos*), sheep (*Ovis*), goat (*Capra*)*,* horse (*Equus*) and pig (*Sus*). All these non-native domesticated animals disappear after the crannog abandonment except a few sporadic occurrences. The conservative bioinformatic approach taken here (see methods), and the typical increase from one or two repeats and low read numbers, to presence in all 8 PCR repeats and high read numbers (200–26,000), indicates the presence of the taxa in the close vicinity and/or at high abundance, on the crannog itself. This is supported by probability modelling using the Bayesian occupancy modelling approach^15^ (Supplementary Information Fig. 3) for each species, which shows that for cattle, deer, sheep, horse and pig the probabilities of occurrence jumps from very low to nearly 1 during site occupation, and in most cases falls again towards the end of the crannog period.

**Figure 3:**
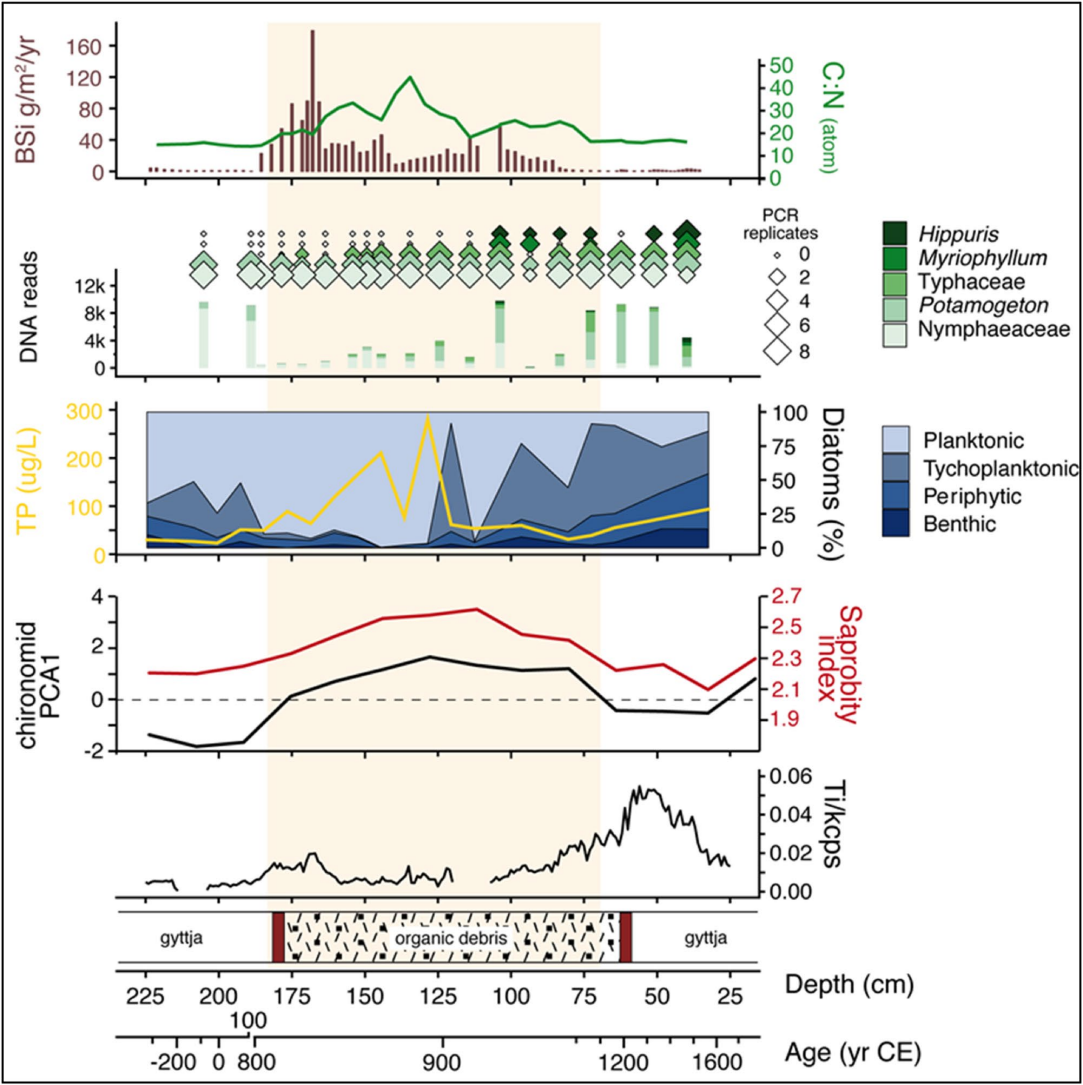
High-resolution analysis of the aquatic indicators in the near-crannog core including biogenic silica (BSi) flux, C:N ratio, *seda*DNA of aquatic plants, major aquatic diatom groups (%) with diatom-based total phosphorus (TP) estimate, saprobity (organic matter water content) index and PCA axis 1 based on chironomids, and titanium (Ti) and core stratigraphy.

### Lipid biomarkers

In order to cross-validate the sedaDNA results we conducted bile acid analyses of the same sediments. The cross-validation comes from the fact that the bile acids can only produced by living animals, including humans, or animals butchered off-site but immediately brought onto site but and not by long-dead or preserved tissues. The bile acids show elevated levels associated with the early crannog phase and some correspondence with the sedaDNA of the domesticates, especially cattle (Fig. 2b). The deoxycholic acid (DCA):lithocholic acid (LCA) ratios indicate faecal matter from human/horse (0.6–4.5) and cattle/sheep/goats (> 5)^16^. It is likely that human/horse was the source at several levels during the crannog phase, based on (i) the low DCA:LCA ratio, and (ii) the peak in chenodeoxycholic acid (CDCA) (52 mg/g/yr) at 114 cm (862–954 CE), which is specific for human or horse. The lack of hyodeoxycholic acid (HDCA) also suggests that pig was not a significant presence on the crannog, corresponding with its low and sporadic presence in the sedaDNA and its common occurrence as a contaminant although not apparently in this case (Fig. 2, Supplementary Information Table 4).

### Other palaeolimnological proxies

Both sedaDNA and lipid biomarkers can also be compared with other indicators, particularly palaeoecological proxies such as pollen, spores, diatoms, chironomids, and geochemical indicators such as XRF, biogenic silica (BSi), and C:N ratio. The pollen diagram from the northern crannog core shows a clear reduction in the principal trees; birch (*Betula*), alder (*Alnus*), oak (*Quercus*) and hazel (*Corylus*) coincident with the crannog-related unit and a rise in grasses (Poaceae), daisy family (Asteraceae), and ribwort plantain (*Plantago lanceolata*), and the appearance of the cultivars oats (*Avena*) and barley (*Hordeum*) type. The micro-charcoal starts at the base of the crannog unit and sustains high influx values throughout the crannog phase, suggesting the use of hearths on-site (Fig. 2a, Supplementary Information Fig. 4).

**Figure 4:**
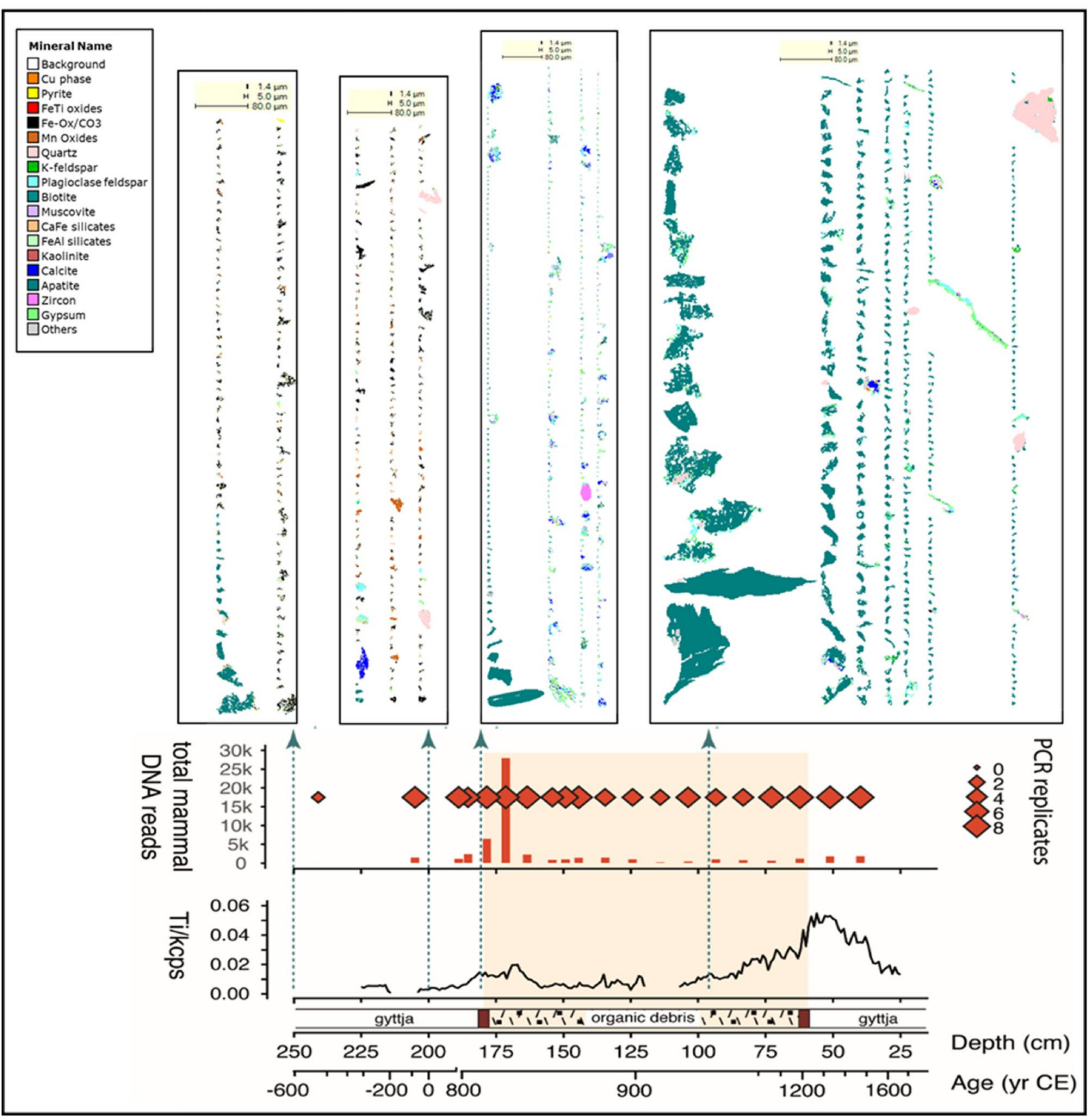
Core Ti and sedaDNA from the northern crannog with mineral grains determined by QEMSCAN at four depths in the core. Note all scale bars are 80 μm.

SedaDNA data of aquatic plants show an increase during crannog use (Fig. 3); the appearance of bulrushes (Typhaceae), mare’s tale (*Hippuris*), and watermilfoils (*Myriophyllum*) which are associated with apparent lake shallowing and eutrophication. This is supported by the BSi curve and diatoms, which show distinctive changes during the crannog phase (Fig. 3), particularly an increase of nutrient-indicating planktonic taxa (*Aulacoseira granulata, Cyclostephanus dubius*) that block out light at the expense of benthic diatoms (e.g., *Aulacoseira subarctica*). The diatom-inferred phosphorus reconstruction shows a significant nutrient increase during the early period of the northern crannog use before falling to previous levels (Fig. 3; Supplementary Information Fig. 5) and the high nutrient input from terrestrial sources is clearly visible in the high C:N ratio. Remains of chironomid larvae (non-biting midges) show an increase in taxa associated with decomposing organic matter, or ‘saprobity’ (Fig. 3) also observed at Neolithic lake village sites in Switzerland^14^. Macrophyte-associated chironomid taxa increase during the crannog phase as do eutrophic/littoral chironomid taxa (Supplementary Information Fig. 6), indicating lake shallowing. Given that the lake outlet is on bedrock, this apparent ‘lake-shallowing’ can only be caused by the creation of a ‘shore-edge’ as the crannog is constructed^17^. Values of Ti, taken as an indicator for in-wash of mineral particles, show an increase, compared to background values, at the start of the crannog phase, concomitant with changes in other proxies.

**Figure 5:**
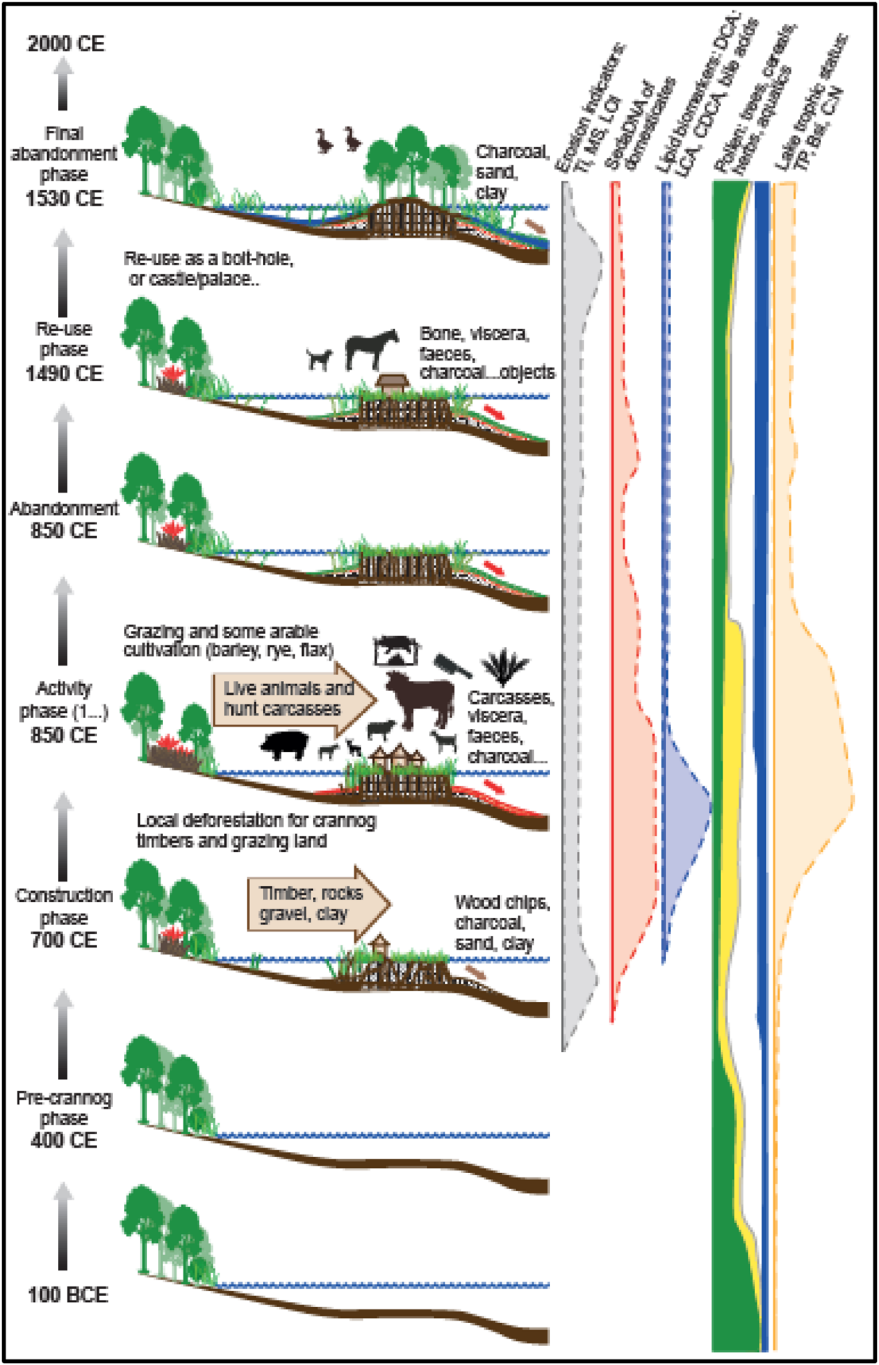
A diagrammatic representation of the chronological phases and multi-proxy results from the crannogs at Lough Yoan, including clastic proxies (MS is magnetic susceptibility), sedaDNA, lipid biomarkers, pollen and lake trophic status indicators .

An additional core taken adjacent to the crannog on the south side of the lake shows a similar pattern in pollen data, with a fall in deciduous trees and rise in herbs and cultivars—including both Poaceae and cereal type pollen (Supplementary Information Fig. 7) with unusually high ribwort plantain (*Plantago lanceolata*) reaching over 20% total land pollen (TLP). This sequence dates with the site construction and occupation and so can be seen as part of the development of the site. A further core through the southern crannog revealed even higher levels (up to 40% TLP) as well as unusually high numbers of bracken spores and sporangia; many of these are on the point of spore-release, indicating summer bracken collection. The spore *Diporotheca webbia*^18^ which is associated with the maiden fern (*Thelypteris*) was also found to peak just above the artificial crannog deposits—probably due to revegetation of the abandoned island. Coprophilous fungal spores peaked in the upper units (habitation floors) of the crannog deposits and a peak in the human or pig whipworm (*Trichuris*) indicates faecal material (Supplementary Information Fig. 7).

### QEMSCAN mineralogy

The automated particle mineral results shown here (Fig. 4) show clear differences between selected horizons in the northern crannog core. In the lowest sample (250 cm, or 648 ± 116 BCE) there is a wide range of minerals present dominated by quartz, plagioclase feldspar, Ca Fe silicates, Fe Ti oxides and chlorite as small particles, with very little apatite (0.08%). A sample at 200 cm from before site construction has a narrower range of minerals but with a few apatite particles. This is also the case with a sample from the beginning of the site construction (187 cm or 838 ± 44 CE). However, the sample from just before site abandonment (95 cm c. 925 CE) shows a mineral assemblage dominated by larger grains (20-30 μm) and higher quantities of apatite (3.67%) (Fig. 4). These large particles of apatite are typically porous with fresh fractured edges. Given the lack of another apatite source (the geology is carboniferous limestone under thick moraine) it is inferred that these are bone micro-fragments derived from on-site butchery.

## Discussion

Our first application of both sedaDNA metabarcoding and lipid biomarkers in a multi-proxy molecular approach to an archaeological lake settlement sequence reveals changes in environment and human activities with unprecedented detail. We reveal complex links between animal husbandry, food resources, crannog use and early anthropogenic impacts on lake ecosystems. An obvious indication of human activity is the high Ti after the start and at the end of the crannog phases, representing influx of mineral material (clay/silt) are interpreted as construction of the structure and its subsequent erosion. The sedaDNA from the northern crannog and the pollen from both crannogs show that the crannogs are constructed in a partially deforested landscape (first century BCE to fourth century CE) but is associated with a further reduction in trees in the ninth century CE with an increase principally in grassland but also in heathland. Given that; lake sedaDNA cannot be leached from the surface, sedaDNA records of vascular plants are associated with the areas very close to the lake shore^19^, the small size of the catchment and the strength of the pollen results, all suggests the signal is derived from a clearance very near to the crannog to form an agricultural hinterland of fields close to the site. This narrative is supported by the bile acids and mammalian sedaDNA which suggest regular animal stocking and butchery activity as supported by the apatite (bone) mineral assemblage.

The strength of the sedaDNA results for domesticates, including cereals, flax and animals, again, indicates that the source of sedaDNA is very close to the core site. Indeed, the sedaDNA results here for mammals (both in reads and replicates), particularly domesticates, are stronger (in repeats) than in most other lake studies although the reads are in line with, but occasionally above, other lake studies^20–23^. A possible explanation for this is the presence of microscopic fragments of apatite (bone). The extraction protocol did not use a homogenisation step with beads (as is common practice) and so the samples would have contained variable numbers of apatite fragments containing or carrying mammalian DNA, and this bone would also have been accompanied by blood and viscera, all resulting from animal slaughter and butchery. This material, particularly stomach contents, may also have been responsible for the elevated bile acid levels surrounding the site.

The remarkably strong sedaDNA and biomarker evidence presented here has several implications of archaeological importance that verify the high potential of our approach. The DNA evidence for barley and oats accords with the major crops grown in early medieval Ireland ^24^. Flax, which is also known from pollen records, may have been processed and woven on the site^12^. From the analysis of plant macrofossils, pollen and coleoptera it had previously been hypothesised that a crannog in Lough Kinale, Central Ireland was used for the processing and/or storage of cereals^9^ and this is validated here from both the sedaDNA and pollen data as well as by the finds of quernstones on both crannogs (Table S1). It is proposed here that the sedaDNA from domesticated animals (cattle, sheep, goats and horse) and red deer was derived from faeces (bile acids), slaughtered carcases and bone from the island. This is in line with excavations of early Irish Medieval crannogs that have produced large quantities of bone including Ballinderry I and II, Lagore, Lough Faughan, Moynagh and Sroove^25^. Indeed, some sites produced vast quantities of bone (e.g. 20 tons from Lagore and 850 kg from Ballinderry I), with over half of the bone from Lagore coming from outside the palisade as did most at Ballinderry, This is taken to probably reflect feasting activity^25^ and shows that the practice of throwing parts of the carcasses off the crannog into the surrounding water was common. At these sites, the anatomical bone assemblage (typically all parts of the skeleton), the age and the butchery marks all point to o-site slaughter and butchery for feasting. These assemblages are similar to Lough Yoan north crannog with a dominance of cattle, followed by sheep and possibly pig, with small amounts of horse and red deer and again possibly goat^25^. The occurrence of horse DNA at Lough Yoan may also reflect its consumption (as at Sroove), whilst the occurrence of red deer is thought-provoking as this was a prestige food closely associated with cattle in the early medieval mind^26^. The bile acid data supports the presence of these domesticates as live animals along with faecal material from human sources. The keeping of live animals on the crannogs is further evidenced by bracken, traditionally used for bedding and as part of the crannog flooring^27^. A Romano-British period crannog from southern Scotland revealed over 1 m of fern (bracken) at its base^28^, whilst underwater excavations of Oakbank crannog in Loch Tay revealed layers of bracken laid down, probably as flooring^29^. Coprophilous fungal spores and the unusual amounts, and pollen clusters of, ribwort plantain pollen, were most likely from sheep/cattle/goat faeces or stomachs. Sheep seem the most likely, as they are known to produce pastures rich in plantains due to nitrogen enhancement and moderate disturbance^30^. The addition of nutrients from the crannog into the lake, and particularly nitrogen and phosphate, reflected in the eutrophic indicators (Figs. 3 and 5), is also the cause of the eutrophication of many Irish and Scottish lakes and wetlands^9^.

These activities, from crop storage, animal slaughter and consumption are all key aspects of worth, value and status in the Medieval world^31^ which also increasingly become aspects of a performative display of wealth and power^13^. Whilst we can rarely know exactly who built these islands, the finds, such as the Medieval Ulster Coarse (MUC) pottery on the southern crannog, suggest the builders and users were the local Irish elite. The dates from both the crannogs and the marginal cores indicates that the islands were contemporary and constructed in the first half to the middle of the eighth century CE, finally going out of use in the tenth century CE with a short re-occupation in the fifteenth-sixteenth centuries CE. In this context the location of Lough Yoan may be pertinent as during the early Medieval period the nearby River Erne formed a contested boundary between three chiefdoms—with regular inter-clan conflict, a situation that was only resolved in 1208 CE, and renewed unrest around the adjacent town of Enniskillen in the fifteenth century CE. (Supplementary Information Fig. 1).

The sedaDNA, bone fragments and bile acids provide direct confirmation of previous inferences from animal bone assemblages that stock was kept, and slaughtered, on crannogs^32^. The slaughter and subsequent feasting probably had both functional (disposal of waste, allowing habitation) and cultural (feasting) associations, and possibly even political significance, as is common in European medieval societies^33^. The plant sedaDNA results also suggests that the island was associated with the storage and processing of arable crops, particularly barley, rye and flax, which is consistent with the quern stones found at both sites. Cultivation would also have taken place around the lake—as indicated strongly by sedaDNA and, although present, less pronounced in the pollen data. Flax retting, a messy and smelly process, could have taken place off the edges of the crannog in readiness for linen fibre production. The most likely practice is both the storage and processing of these crops in a location safe from theft or pilfering, although it may well also have had higher social significance^13^.

This study reveals the remarkable quantity of novel archaeological information that can be recovered by a core through the lacustrine biogeochemical halo that surrounds an archaeological site within or at the edge of such lake or wetland sites (Fig. 5). Not only does this zone preserve a sedimentary record and macro and microfossils, but also the sedaDNA and bile acids derived from both plants and animals. The correspondence of the sedaDNA, lipid biomarkers and pollen with the stratigraphy and immobility of both particulate and molecular proxies in lake sediments invalidates modern contamination (but see Supplementary Information Methods Text). The resulting environmental sequence provides a much more comprehensive chronology for the site than can be gained by the dating of exposed timbers alone as it includes construction, use, re-use and abandonment. Although this can be partially achieved using stratigraphy, AMS dating, XRF, and traditional palaeolimnological proxies like pollen and invertebrates, it is the integration of sedaDNA and lipid biomarkers that produce direct evidence for the on-site presence of animals and plants. This creates an activity profile for the site and its change of use during its life-time. Further work on combining sedaDNA with lipid biomarkers offer the possibility of determining species and even human intensity of use. These techniques will be practical for many other wetland sites around the world where the site is surrounded by waterlogged sediments spanning the period of use of the site. This approach can provide a step-change in our understanding of waterlogged or submerged wetland villages, moated sites and lake settlements, which are notoriously difficult to excavate. This has wide-ranging implications across archaeology, such as (1) capturing valuable insights into prehistoric humans, such as those with transient lifestyles, which have no identifiable associated archaeological remains; (2) advancing archaeological understandings gained through excavation, such as providing independent anthropic chronologies that can refine the timings of human occupation and activity; (3) providing the opportunity to quickly and non-destructively characterise a larger number of archaeological sites than is possible through excavation.

## Methods

### Study site and sampling

The site lies in the Lower Erne valley just 0.4 km from the river Erne and 4 km upstream of the Lower Lough Erne on the outskirts of Enniskillen. The site was selected to be suitable for detecting archaeological activities in the lake catchment by being a small lake (Lough Yoan 0.11 km^2^), in a small catchment (0.48 km^2^) with a catchment to lake ratio of 4.3), which contained two crannogs but no other significant archaeology in the catchment (Supplementary Information Fig. 1). The crannogs were also constructed within 30 m of the lake shore. Cores were taken 10–15 m into the lake adjacent to both crannogs on the open lake side, and through the southern crannog (Fig. 1 Supplementary Information Table 1). Both crannogs are reflected in the stratigraphy with typical organic lake gyttja being augmented by wood fragments (some worked), sand, grit, charcoal, nuts, seeds, and bone fragments (some burnt). This stratigraphy thickens towards the island and is generally overlain by lake-gyttja deposited after crannog abandonment. XRF also shows that there are typically peaks in clastic sediment represented by Ti and Si coinciding with this stratigraphic horizon at the base and the top of the unit which could be traced directly to the island. The cores were 10 cm diameter and taken using a modified Nesje piston corer from a coring raft. The corer head was dowsed with exotic DNA (pineapple) which was not detected in any samples. Cores were transported and stored at 4 °C. Samples were taken from the innermost 1 cm of the core after core splitting in a clean laboratory.

### Determining a site chronology and sediment geochemistry

Dating is provided by 27 ^14^C AMS dates determined at the University of Oxford Radiocarbon Laboratory (Supplementary Information Table 2). OxCal version 4.4^34^ was used for calibrating radiocarbon dates with the IntCal20 curve^35^ and for creating age-depth models (Supplementary Information Fig. 2). SedaDNA and faecal biomarkers were extracted from a parallel core taken within 2 m of dated core adjacent to the northern crannog. Both cores analysed for sedXRF and were correlated using Ti and Fe element data. Clear peaks in Ti and Fe available in both cores were used as tie points which were then used in software package QAnalySeries^36^ to correlate these cores. This information was then used to transpose the age model of the dated core onto the correlated depths of the core used for sedaDNA/biomarkers. For geochemistry core XRF scanning was undertaken using an ITRAX XRF scanner^37^ directly on the split cores scanned at 2 mm resolution using 30 kV, 30 mA settings and a 15 s count time at the British Ocean Sediment Core Research Facility (BOSCORF) at the National Oceanography Centre, Southampton (NOCS). In total 39 elements were identified including lithogenic indicators (Si, Al, K, Ti, Zr, Rb) and anthropogenic and heavy metal indicators (P, Ca, Cr, Zn, Ba, Sr, Pb). The resultant elemental intensities, measured in counts per second (cps), were vetted to remove unreliable results which occurred at the boundaries of samples and in particularly coarse sediment horizons. Following data control analysis, the remaining elemental relationships were plotted as ratios to Ti as the conservative detrital element.

Automated SEM-EDS^38^ analysis was carried out using QEMSCAN technology. QEMSCAN is an automated mineralogical analysis system based on a scanning electron microscope that provides rapid determination and quantification of the mineralogy, chemical composition, grain size and shape of a variety of sample types. Data collection is operator independent, with the acquisition of very large data sets, hence the results are statistically reliable and provide highly reproducible mineralogical analyses. The technology has been widely used in the analysis of soil and sediment samples^39^. There are various modes in which the QEMSCAN can be run; including particle mineral analysis (PMA) mode where individual particles are mineralogically mapped, bulk mineral analysis (BMA) mode where the bulk mineral assemblage of a sample is very rapidly determined, field image mode where the entire sample is mineralogically mapped and trace mineral search (TMS) mode where only particles with a specified backscatter electron threshold are mapped^38^. In this study the samples were analysed using the PMA measurement mode using a 1.5 µm beam stepping interval with ~ 50,000 mineral grains measured. The raw data acquired during QEMSCAN analysis is based upon classification of the individual X-ray spectra by using a look-up table containing in excess of 600 known mineral phases and chemical compositions. Spectra that cannot be matched to known phases at this point are classified as “others” and the coordinates stored (they can be assigned to a chemical phase at a later date). The raw data are then processed by assigning similar pixel types to single categories which may be a mineral phase, chemical composition or any other category. In this way, edge and boundary effects can be accounted for and any other phases present with a discrete chemical composition can be assigned to a distinct phase category.

### SedaDNA metabarcoding

DNA was extracted from 19 samples and three negative controls at the ancient DNA (aDNA) dedicated laboratories at the Laboratoire d’ECologie Alpine, University Grenoble Alpes (LECA) from the Northern crannog core. Metabarcoding was chosen as the target was vascular plants and mammals, and as high taxonomic precision as possible (for more explanation see Supplementary Information DNA Methods Text). For each sample, we extracted DNA from ~ 15 g of wet sediment using the Macherey–Nagel Kit (Düren, Germany), following the manufacturer’s instructions. All PCRs were performed in an *a*DNA dedicated room, using the g and h universal plant primers for the short and variable P6 loop region of the chloroplast trnL (UAA) intron and including a unique 8 bp long flanking sequence (tag) at the 5′ end to allow parallel sequencing of multiple samples^19,40^. For mammal DNA amplification, the MamP007F mammal primer was used for a 60–84-bp fragment of the mitochondrial 16S gene^20^. DNA amplifications were carried out in 50 μL final volumes containing 5 μL of DNA sample, 2 U of AmpliTaq Gold DNA Polymerase (Life Technologies, Carlsbad, CA, USA), 15 mM Tris–HCl, 50 mM KCl, 2.5 mM MgCl_2_, 0.2 mM each dNTP, 0.2 μM each primer and 8 μg Bovine Serum Albumin. Three PCR negative controls were also carried out. All PCR samples (DNA and controls) were randomly placed on PCR plates. Following the enzyme activation step (10 min at 95 °C), PCR mixtures underwent 45 cycles of 30 s at 95 °C, 30 s at 50 °C and 1 min at 72 °C, plus a final elongation step (7 min at 72 °C). Eight individually tagged PCR repeats were made for each sample to increase the chance of detecting taxa represented by low quantities of DNA, as well as to increase confidence in the taxa identified. Equal volumes of PCR products were mixed (15 μL of each), and 10 aliquots of 100 μL of the resulting mix were then purified using MinElute Purification kit (Qiagen GmbH, Hilden, Germany). Purified products were then pooled together before sequencing; 2 × 100 + 7 paired-end sequencing was performed on an Illumina HiSeq 2500 platform using TruSeq SBS Kit v3 (FASTERIS SA, Switzerland).

Sequence data were analysed using the OBITools software package^41^. First, direct and corresponding reverse reads were assembled using *illuminepairedend*, and sequences having a low alignment quality score (threshold set at 40) were filtered out. The retained reads were assigned to relevant samples using ngsfilter, keeping sequences matching 100% with tags and allowing a maximum of three mismatches with primers. Strictly identical sequences were then merged together (dereplication) using *obiuniq*, keeping information on their distribution among samples. All sequences with only a single copy and/or shorter than 12 bp were filtered out using *obigrep*. *Obiclean* was then used to identify amplification and sequencing errors, using a threshold ratio of 5% for reclassifying ‘internal’ sequences to their relative ‘head’ sequence^42^. Finally, using the sequences were compared with a the global EMBL database (release r117 from October 2013) by running *ecopcr.* Sequences assigned to non-native taxa were blasted to check for potential wrong assignments (http://www.ncbi.nlm.nih.gov/blast/).

Extreme caution must be taken before accepting a taxonomic assignment in an environmental sample^19,43,44^. To reduce risk of misidentifications, only sequences matching 98% to reference library entries and occurring as at least 10 reads per PCR repeat for plants and 5 reads per PCR repeat for mammals were kept. The following were also removed: (1) sequences having higher frequencies in negative controls than in samples, (2) sequences occurring in < 3 repeats in total (i.e. across all samples), (3) sequences belonging to exotic food plants and thus suspected to be contaminants, and (4) sequences suspected to be droplet contaminants or overflow from samples from another study run at the same time. One complete sample, which appeared as an outlier in terms of low number of reads and repeats, was excluded. By applying these thresholds, rare taxa were possibly missed but potential errors were removed. Our procedures for detecting contamination were rigorous particularly due to the risk from domesticated mammal DNA. We ran a large number of controls (9 with 8 repeats each) and the amplification of domestic animals in these controls was extremely, 0% of controls for most of species, 1.4% for *Ovis* and 6.9% for *Capra hircus* (See Supplementary Information Table 5). In order to take into account the risk of false positives, we estimated species occupancy using a Bayesian site occupancy detection modelling (SODM) to estimate the frequency of false detections^15^ SODM allow to estimate species occupancy and also the rate of false detections while taking into account the possibility of false positives; the frequency of contamination into the controls (upper 99% confidence interval) was included in SODM as prior for the frequency of false positives. Estimates of occupancy, detection probability, and probability of false detection were then used to calculate the risk that mammal occurrence in a given sample may be a false positive. Simulations showed that this approach allows successfully limiting false positives in sedaDNA analyses. Models included an autoregressive term to take into account the temporal autocorrelation of occupancy^15^. We run three MCMC chains in STAN, each with 15 000 iterations; the first 5000 iterations were discarded as burn-in. For all models, r-hat was < 1.1, confirming model convergence.

### Lipid biomarkers

Lipid biomarker analysis for bile acids was conducted following standard protocols based on Bull et al.^45^ and outlined in Mackay et al.^46^. Briefly, 10 μL of androstanol (0.1 mg mL^−1^) and 10 μL of hyocholic acid (0.1 mg mL^−1^) were added as internal standards to each sample of approximately 1 g of dried, homogenised sediment. Total lipids were extracted with solvents (DCM:MeOH, 2:1, v/v) using microwave assisted extraction (heated to 70 °C over 10 min then held at 70 °C for 10 min; saponified using 5 M sodium hydroxide in MeOH and separated into neutral and acid fractions using aminopropyl SPE columns. The acid fraction was methylated using 3 mL of trimethylsilyldiazomethane (TMS-DAM) in toluene/methanol (4:1 v/v). Silica gel column chromatography was used to isolate the hydroxylated carboxylic acid fraction (containing the bile acids) from the methylated acids. Methylation of the acid fraction was achieved using 3 mL of trimethylsilyldiazomethane (TMS-DAM) in toluene/methanol (4:1 v/v). The bile acids were derivatised using 30 μL of BSTFA + TMCS (99:1 v/v) and dissolved in 50–100 μL of ethyl acetate prior to gas chromatography-flame ionization detection (GC-FID) and gas chromatography-mass spectrometry (GC–MS) analysis. GC–MS analyses were performed on an Agilent 7890B GC injector (280 °C) linked to an Agilent 5977B MSD (electron voltage 70 eV, source temperature 230 °C, quad temperature 150 °C multiplier voltage 1200 V, interface temperature 310 °C) in full scan mode (50–600 amu/sec). Separation was performed on an Agilent fused silica capillary column (HP-5, 60 m × 0.25 mm ID × 0.25 um df), with Helium as a carrier gas. The sample (1 μL) was injected in splitless mode (1 min splitless time). Bile acid derivatives were analysed using the following temperature programme: 40 °C (held for 1 min) to 230 °C at 20 °C min^−1^ then to 300 °C at 2 °C min^−1^ and held for 20 min. GC–MS peaks were identified through comparisons with known mass spectra (NIST08) and standards where possible. Analytes were quantified based on internal standards. The dominant faecal matter source was identified using the ratio of deoxycholic acid (DCA) to lithocholic acid (LCA) ratio^16^. Based on modern experimental data, the values of this ratio can be ascribed in the following way: < 0.4 pigs and/or geese; 0.6–4.5 humans and/or horses; > 5 ruminants (cattle, sheep and goats)^16^. Whilst the dominant faecal source can be identified using these ratios, this does not preclude the presence of other faecal sources in smaller quantities.

### Pollen and spores, diatoms, chironomids

Standard extraction methods were used for pollen and spores, involving acetolysis and hydrofluoric acid digestion, the addition of an exotic marker (Lycopodium) and mounting in Si oil. Standard keys were used for identification along with the reference collections at the University of Southampton and the Pollen Laboratory and the University of Plymouth. The raw data is in Supplementary Information (Excel File Raw Pollen Data Supplementary Dataset 2). Diatom samples were prepared by digestion with hydrogen peroxide (H_2_O_2_)^47^, after which they were mounted on microscope slides using Naphrax. At least 400 diatom valves were counted at × 1000 magnification using a Nikon Eclipse 80i microscope. Images of selected frustules were captured using the Nikon Digital Sight DS-L2 and Nikon DS-Fi1 microscope attachments. Identification of the frustules follows standard guides particularly Krammer^48^. Chironomid samples were soaked in 10% potassium hydroxide (KOH) at 70 °C for 30 min, then sieved using a 90 µm mesh sieve. Head capsules were mounted on permanent slides using hydromatrix and identified at 100–400 × magnification. Identification of head capsules follows Brook et al.^49^. Saprobity index is calculated following following^50,51^. All statistical analyses were run in R using vegan, analogue and rioja packages for R. For the diatom TP transfer function^52^ the combined European training set (craticula.ncl.ac.uk/Eddi), which is a very broad training set with a reasonable fit to the fossil data.

## Supplementary Information

Supplementary Information.

Supplementary Dataset 1.

Supplementary Dataset 2.

## Data Availability

The full processed sedaDNA file is available online from EBI-ENA under Project No PRJEB45292 (ERP129375) with the look-up tag file given as a Supplementary Information Dataset 1 (Sample_tag_primer_Crannogs2 LYN.xls).
