# Supplementary material for: Ancient DNA, lipid biomarkers and palaeoecological evidence reveals construction and life on early medieval lake settlements
Source: Sci Rep. 2021 Jun 3;11:11807. doi: 10.1038/s41598-021-91057-x (PMC8175756; doi:10.1038/s41598-021-91057-x)
Supplement: Supplementary file 3 — Supplementary Dataset 2. [file 41598_2021_91057_MOESM3_ESM.pdf]

Ancient DNA, Lipid Biomarkers and Palaeoecological Evidence Reveals Construction and Life on early Medieval Lake Settlements

Supplementary Information

A.G. Brown*, M. Van Hardenbroek, T. Fonville, K. Davies, H. Mackay, E. Murray, K. Head, P. Barratt, F. McCormick, G.F. Ficetola, L. Gielly, A.C.G. Henderson, A. Crone, G. Cavers, P. G. Langdon, N. J. Whitehouse, N. J., D. Pirrie, I. G. Alsos
Corresponding author. Email: antony.g.brown@uit.no

This PDF file includes:
Figures 1-7
Tables 1-5

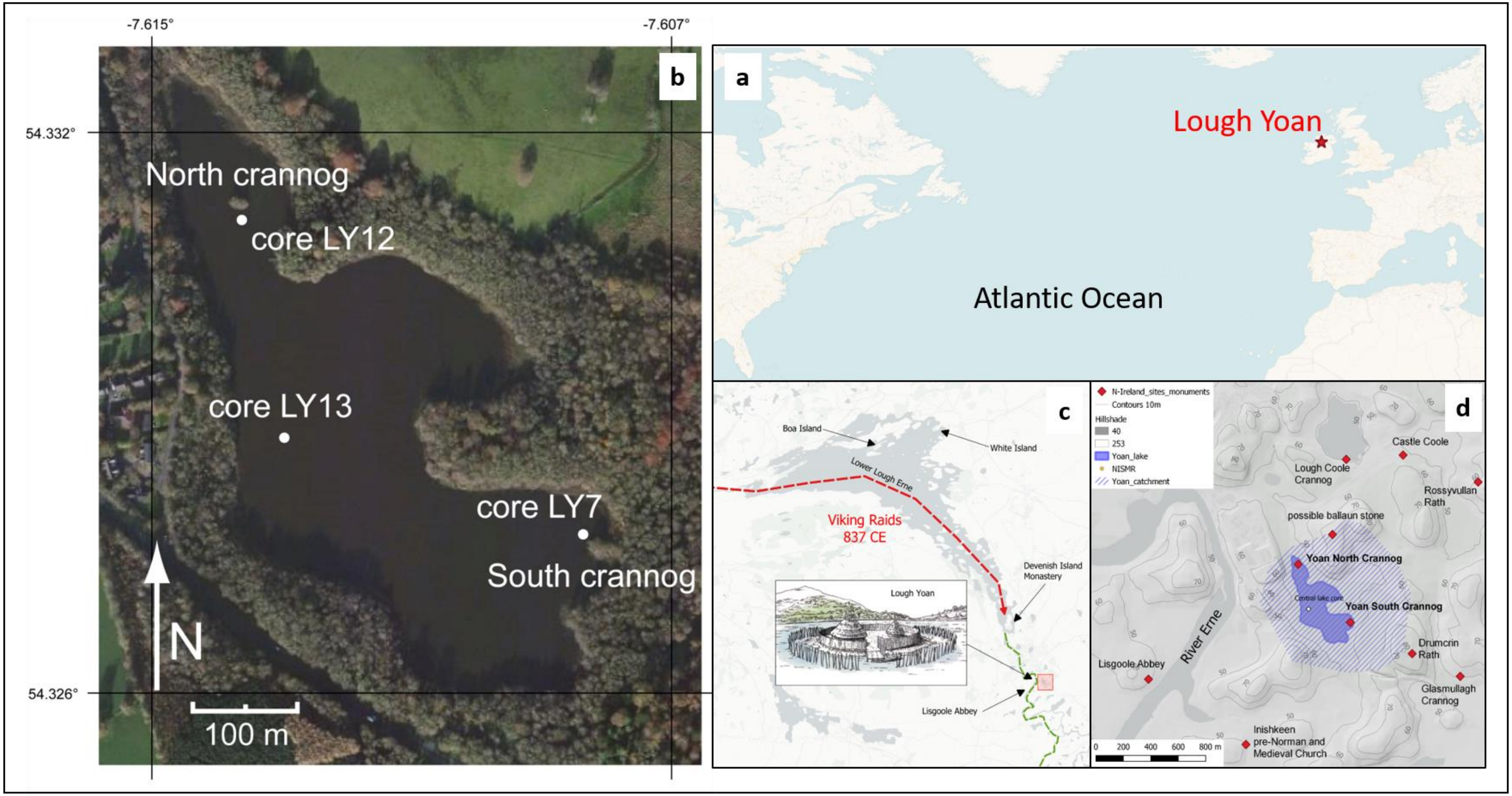

Fig. 1 . a) site location in northern Ireland, b) the Lough Yoan with coring sites mentioned in the text, c) location in the Upper Lough Erne system, Viking raid (837 CE) and clan boundary, d) archaeological sites in the surrounding area and Lough catchment. Fig. 1b used material under Copyright 2021 Google Image Landsat/Copernicus Data S1O. NOAA.U.S.Navy. NGA. GEBCO used under Creative Commons License 4.0

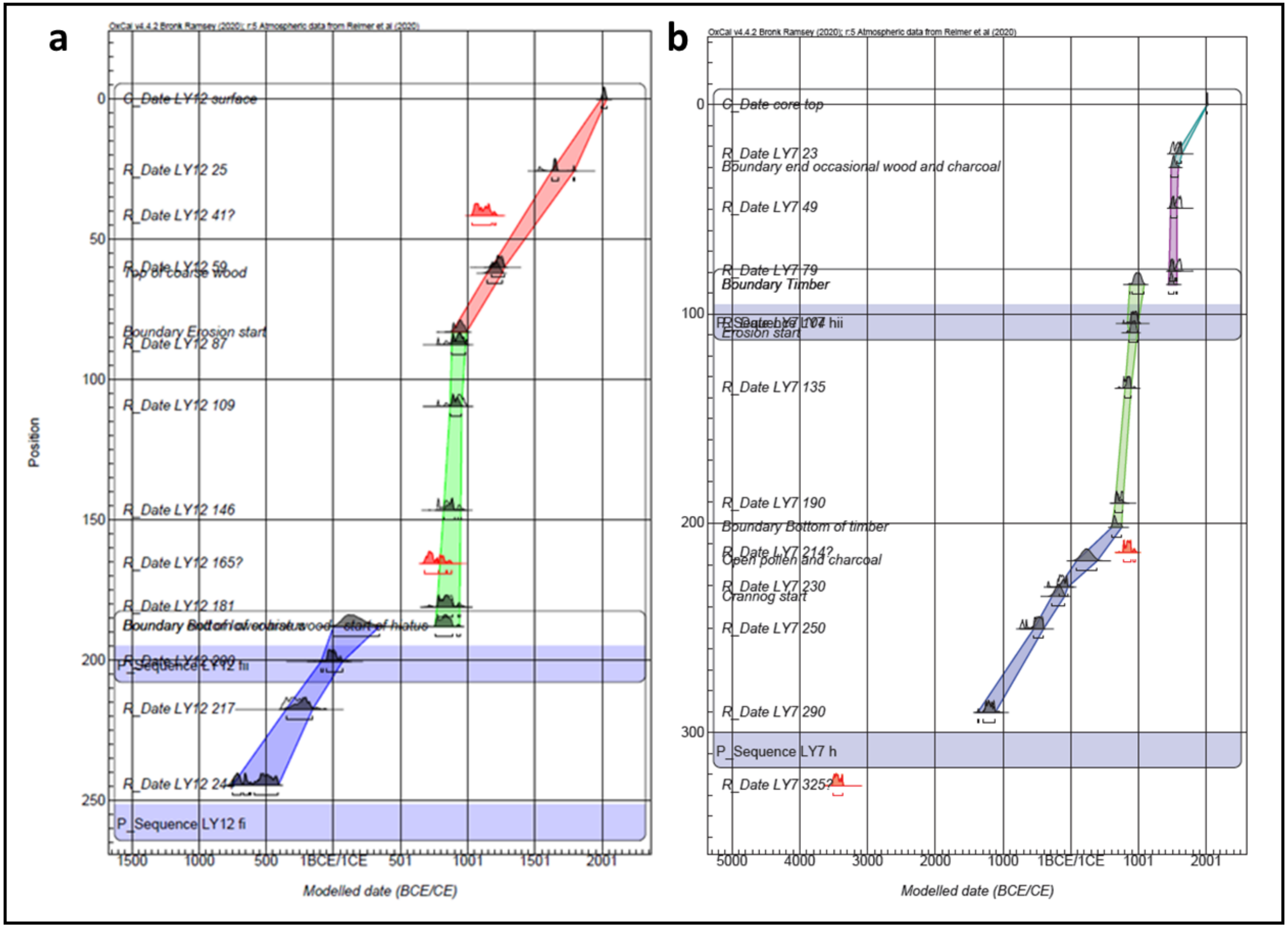

Fig. 2(a) Preferred OxCal model for northern crannog. (b) OxCal model for southern crannog with hiatus and medieval re-occupation, dates in red are excluded from the model

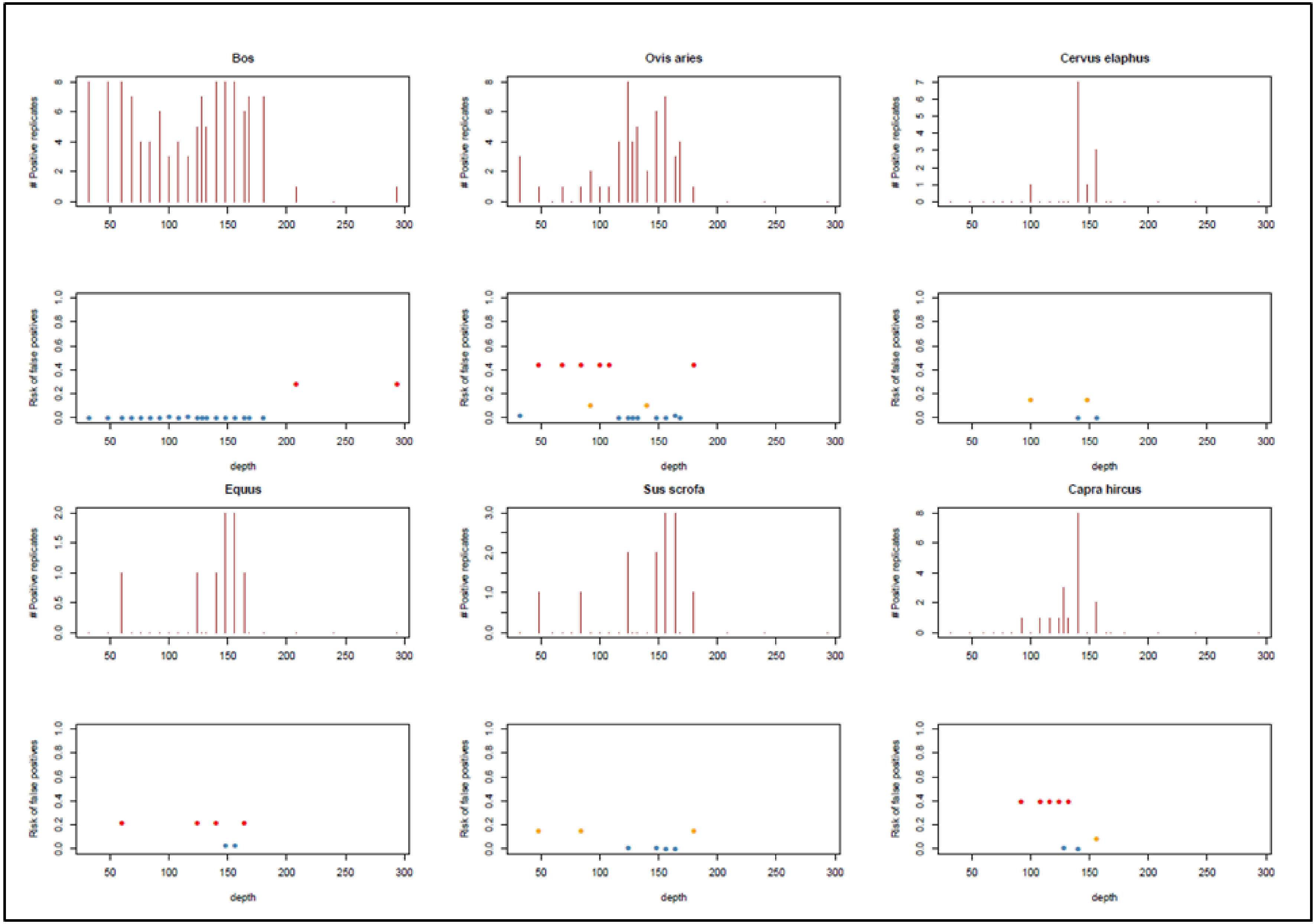

Fig. 3. Mammal sedaDNA metabarcoding reliability modelling: the CAR-SODM approach ([15]) for selected species.

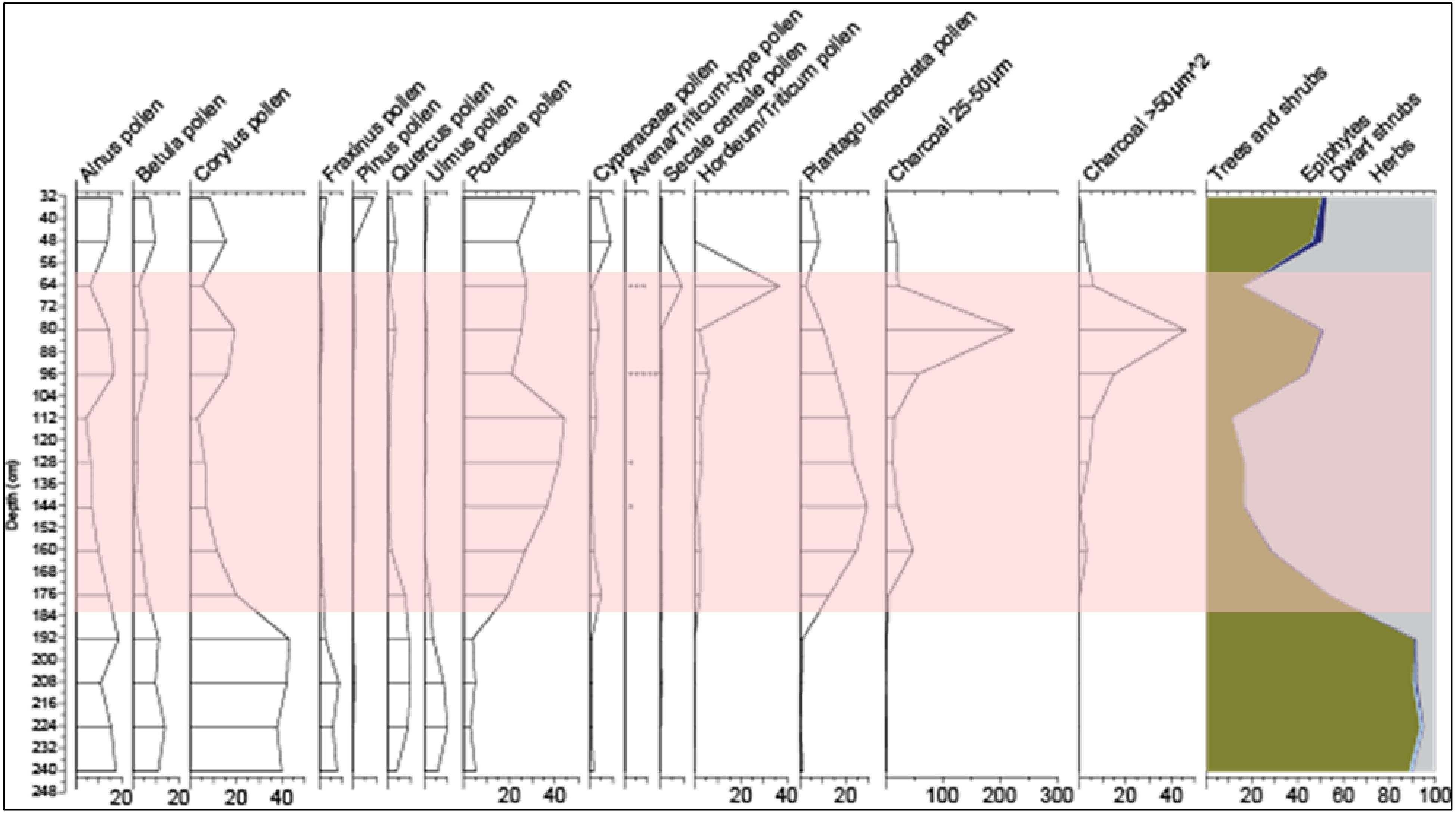

Fig. 4. Summary pollen diagram for Northern Crannog lake core (LY12). Pink shading covers the levels that accumulated during the crannog period.

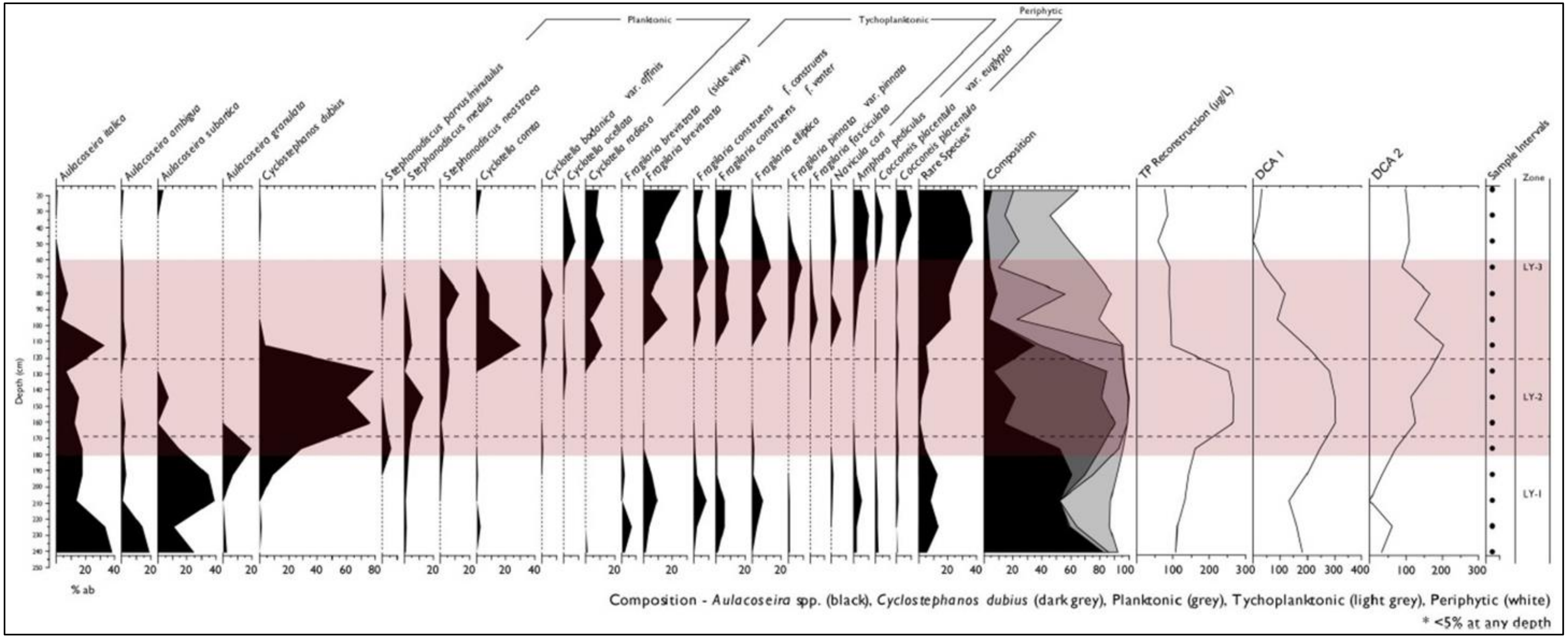

Fig. 5. Summary diatom diagram for northern crannog lake core (LY12). Pink shading covers the levels that accumulated during the crannog period.

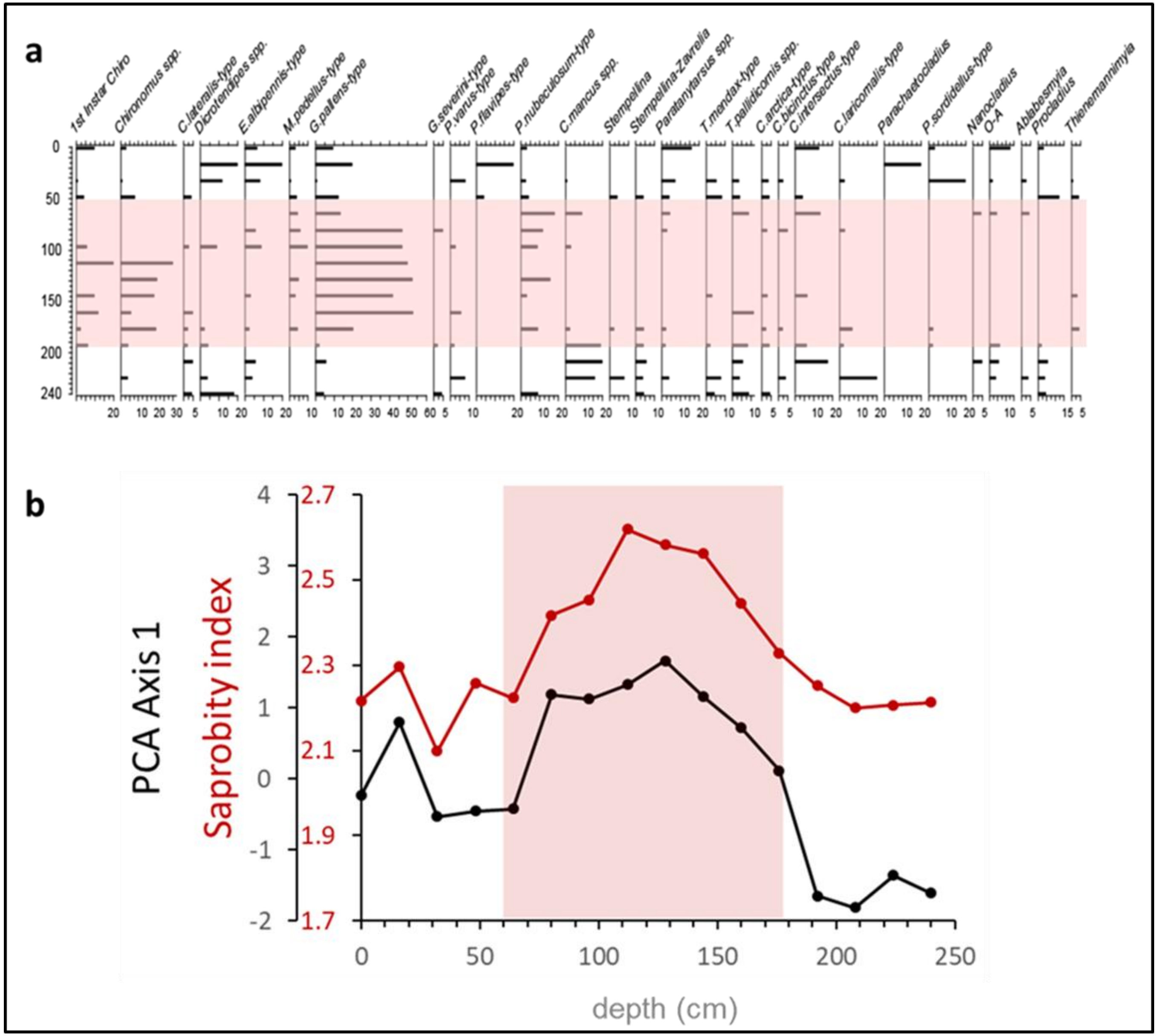

Fig. 6. Summary chironomid diagram for southern crannog lake core (LY12). Pink shading covers the levels that accumulated during the crannog period, (b) the saprobility index reconstruction alongside PCA axis 1 scores from chiromomids.

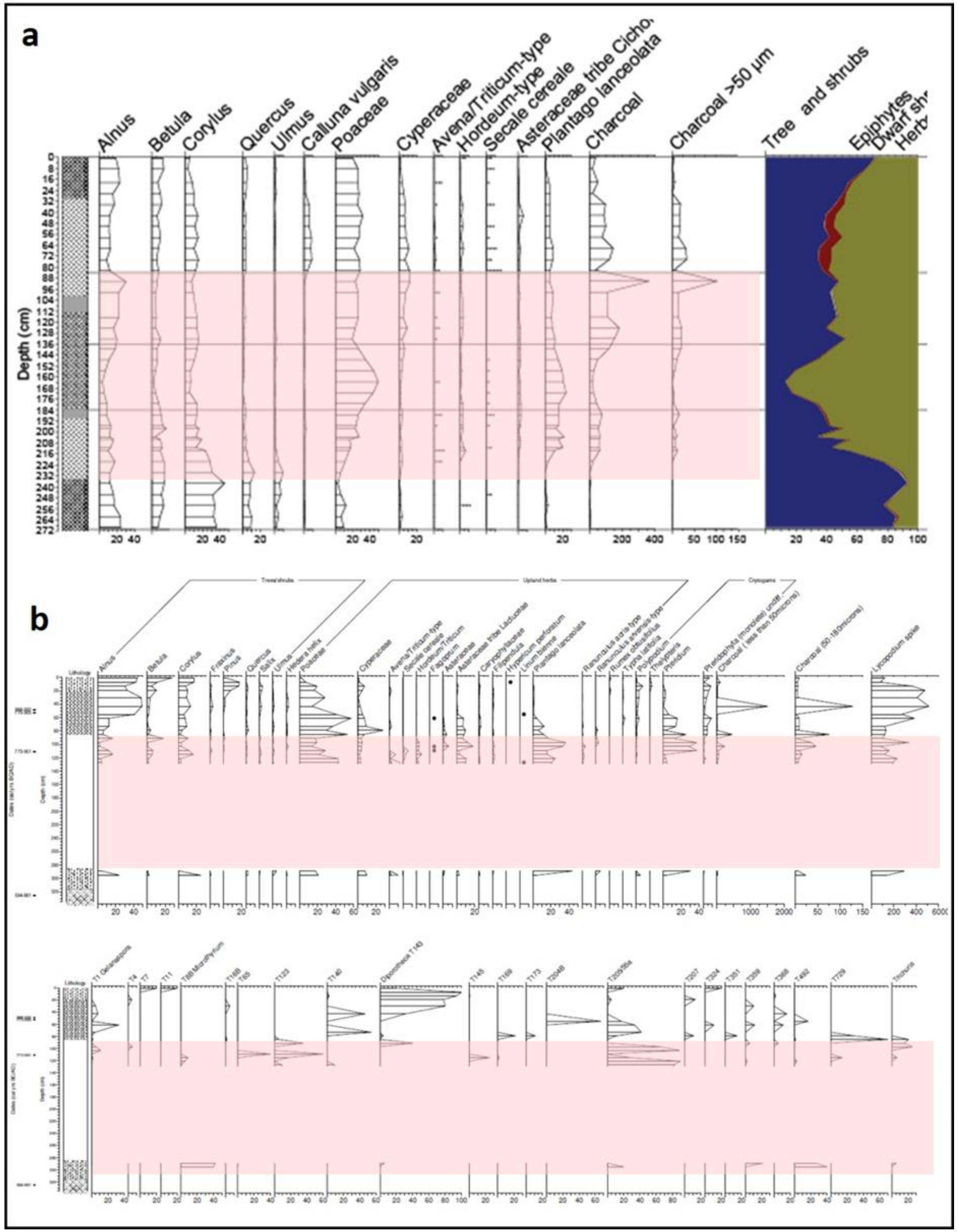

Fig. 7. Selected pollen and spore data from the southern crannog, (a) pollen and charcoal from the core adjacent to the crannog, (b) stratigraphy, 14C dates, selected pollen and NPPs, and Trichuris from the core through the southern crannog. Pink shading covers the levels that accumulated during the crannog period.

Tables 1-4

Location	Crannog	Island diam. (m)	Dist. from shore (m)	Dates	Archaeological material
Yoan (N)<br>Lat:54°19'49.25<br>Long: 7°36'49.31<br>Alt: 51m	Northern crannog 'Gortgonnell/ Killyherlin'	26	30.5	1044-1264 CE (pile)<br>777-994 CE	Whetstones, quernstones (Wood-Martin 1886) animal bone (2 incisor teeth, several fragments of bone, two fragments of ribs and part of a jaw-bone)(FCM_1978_133)
Yoan (N)<br>Lat: 54°19'38.64<br>Long: 7°36'30.63	Northern crannog marginal core LY12	(26)	26	*756-962 CE*	wood chips (this study)
Yoan (S)<br>Lat: 54°19'38.21<br>Long: 7°36'29.46<br>Alt: 52m	Southern crannog 'Drumcrin' core LYSC	24	causeway	*943 CE (887-994)*<br>*870 CE (774-866)*<br>*858 CE (773-963)*	fragment of rotary quern (FCM_1977_057) MUC x 3: decorated rim sherd, plain body sherd, base sherd (DoE), wood chips
Yoan (S)<br>Lat: 54°19'48.73<br>Long: 7°36'49.10	Southern crannog marginal core LY7	(24)	35	*714-1103 CE*	wood chips

Table 1. Descriptive statistics, including archaeology, of the two crannog islands and lake. Dates are calibrated 14C and use coarse woody debris and the start of the Ti rise as the basal crannog date in the cores (see text for discussion of dating criteria) and MUC is Medieval Ulster coarse-ware.

Lab code (ORAU)	Sample code, depth (cm)	Material dated	Ident.	Raw 14C date BP	± (1σ)	$F^{14}C$	± (1σ)	$\delta^{13}C$
43166	LY12 25-26	plant remains	*Pteridium*	260	25	0.9681	0.00303	-28.72
43167	LY12 41-42	seeds	*Rubus*	921	26	0.89165	0.0029	-27.32
41559	LY12 59-61	plant remains	terrestrial leaf fragment	821	33	0.90281	0.00374	-27.86
43168	LY12 87-88	seeds	*Prunus spinosa*	1146	25	0.8671	0.0027	-25.15
42084	LY12 109-110	plant remains		1142	26	0.86746	0.00283	-27.14
43169	LY12 146-147	plant remains	*Pteridium*	1173	25	0.86413	0.00273	-26.13
43170	LY12 165-166	plant remains	mixed plant remains	1249	26	0.85602	0.00275	-28.35

41561	LY12 180-182	plant remains	terrestrial leaf fragment	1188	32	0.86251	0.00342	-26.12
45184	LY12 200	plant remains	monocot stems	2016	26	0.77805	0.00257	-43.16
43171	LY12 217-218	plant remains	cf. Dicranales	2205	45	0.75974	0.00435	-42.33
43172	LY12 244-245	plant remains	cf. Dicranales	2443	28	0.73774	0.0026	-39.60
43159	LY7 23-24	plant remains	cf. *Lemna*	342	24	0.9583	0.00283	-27.23
43160	LY7 49-50	wood	Unid.	344	25	0.95804	0.003	-28.16
43161	LY7 79-80	plant remains	*Prunus spinosa*	354	26	0.95686	0.00306	-26.30
41557	LY7 104-105	plant remains	terrestrial leaf fragment	1120	32	0.86989	0.00349	-27.89
43162	LY7 135-136	wood	Unid.	1195	26	0.8618	0.00281	-30.21
42083	LY7 190-191	plant remains	Unid.	1305	33	0.8501	0.00346	-27.41
45178	LY7 214	plant remains	*Pteridium*	1184	27	0.86297	0.00291	-28.77
43870	LY7 230-231b	sediment	bulk sediment	2122	28	0.76789	0.00263	-32.79
45179	LY7 250	sediment	bulk sediment	2421	31	0.73979	0.00282	-29.90
43164	LY7 290-291	sediment	bulk sediment	2974	29	0.69059	0.0025	-28.62
43165	LY7 325-326	sediment	bulk sediment	4655	32	0.5602	0.00222	-31.57
45180	LYSC 50	wood	wood fragments	1103	26	0.87165	0.00282	-27.39
45180	LYSC 50	wood	wood fragments	1160	27	0.86551	0.00286	-27.34
45181	LYSC 110	plant remains	*Pteridium*	1165	27	0.86504	0.00294	-27.47
45182	LYSC 275	plant remains	terrestrial leaf fragment	841	26	0.90061	0.00293	-28.82
45183	LYSC 325	plant remains	terrestrial leaf fragment	1226	25	0.85845	0.00264	-29.21

Table 2. AMS Dates from, ORA (Oxford) for LY12, LY7 and LYSC.

Event	Date Estimate based on the preferred age-depth models with 95% range and mean age point
**North crannog**	
Crannog establishment with bottom timber (188cm), which also coincides with the opening of the landscape and first increase in charcoal in pollen (in the pollen sample at 184cm)	755-940 CE (95% CI) or 827 CE ± 44 (mean ± SD)
Start of erosion (increasing Ti at 83cm) –*abandonment and end of the use*	883-990 CE (95% CI) or 936 CE ± 31 (mean ± SD)
End coarse woody debris and charcoal (62cm)	1145-1256 CE (95% CI) or 1203 CE ± 29 (mean ± SD)
**South crannog**	
Opening of the landscape (starting after 229cm depth)<br><br>First charcoal in pollen slides (218cm depth)	144 **B**CE – 6 CE (95% confidence interval)<br>61 ± 41 **B**CE (mean ± SD)<br><br>85-390 CE (95% confidence interval)<br>236 CE ± 76 (mean ± SD)
Crannog establishment with bottom timber (202cm depth)	605-751 CE (95% CI)<br>666 CE ± 41 (mean ± SD)
Start of erosion (increasing Ti at 109cm) – *end of first phase of occupation/use*	865-984 CE (95% CI)<br>921 CE ± 33 (mean ± SD)
End of first phase with top timber (86 cm depth) - *abandonment*	871-1084 CE (95% CI)<br>986 CE ± 47 (mean ± SD)
Start medieval reoccupation (from 86cm depth)	1441-1564 CE (95% CI)<br>1490 CE ± 25 (mean ± SD)
End of medieval reoccupation (end of wood and charcoal at 30cm depth)	1480-1587 CE (95% CI)<br>1527 CE ± 26 (mean ± SD)

Table 3. Chronologies from cores for the north and south crannogs

	Bile acid concentrations (μg/g)							
Sample depth (cm)	LCA	DCA	CDCA	HDCA	DCA:LCA	DCA:LCA faecal source*	CDCA faecal source*	Identification of faecal source based on all bile acids
32	0.24	0.83	*0.11*	-	3.45	Human/horse	Human/goat/horse	Human/horse
48	-	-	-	-	n/a			
60	*0.30*	3.47	*0.11*	-	11.39	Cattle/sheep/goats	Human/goat/horse	Dominated by cattle/sheep/goats but mixed source
68	*0.10*	1.75	-	-	17.12	Cattle/sheep/goats		Dominated by cattle/sheep/goats
76	0.58	4.33	*0.10*	-	7.50	Cattle/sheep/goats	Human/goat/horse	Dominated by cattle/sheep/goats but mixed source
84	4.14	-	-	-	n/a			
92	2.23	1.93	-	-	0.86	Human/horse		Human/horse
100	1.60	-	0.52	-	n/a		Human/goat/horse	Human/horse
108	0.57	2.39	-	-	4.22	Human/horse		Human/horse
116	1.03	-	-	-	n/a			
124	0.57	3.59	-	-	6.32	Cattle/sheep/goats		Dominated by Cattle/sheep/goats
128	-	-	-	-	n/a			
132	0.36	2.03	-	-	5.63	Cattle/sheep/goats		Dominated by Cattle/sheep/goats
140	2.81	3.69	-	-	1.31	Human/horse		Human/horse
148	0.56	4.18	-	-	7.44	Cattle/sheep/goats		Dominated by cattle/sheep/goats
156	0.64	6.30	-	-	9.84	Cattle/sheep/goats		Dominated by cattle/sheep/goats
168	0.37	0.88	-	-	2.36	Human/horse		Human/horse
180	*0.06*	0.99	-	-	16.13	Cattle/sheep/goats		Dominated by cattle/sheep/goats
240	-	-	-	-	n/a			
294	*0.07*	0.91	-	-	14.01	Cattle/sheep/goats		

Table 4. Results of lipid biomarkers analysis of northern core (LY12). Values in italics fall below the detection/quantification limit (ca. 0.4 ug/g).

DNA Methods Text: Methodology and contamination procedures.

Metabarcoding was chosen as the target organisms were vascular plants and mammals, and as high taxonomic precision as possible was required to compare with the archaeology of this and other sites. The sedaDNA and lipid biomarkers in lake sediments are known to be fixed soon after sediment deposition[20,53,54,55,56] and are not subject to leaching or vertical movement in the sediment column. This has been specifically tested for sedaDNA in similar lakes in Scotland UK[57]. This also applies to other proxies such as pollen, diatoms and chironomids[58].

A potential problem for this type of analysis is contamination either in the field during extraction, sampling and laboratory analyses. We minimized the risk of contamination in the field by using a wide diameter (10cm) corer which was dosed with exotic DNA (pineapple) which was not detected in any of the samples. The core was also only opened in a clean laboratory and all sampling followed DNA clean procedures in a dedicated aDNA laboratory (LECA, Grenoble). There is also a known risk of contamination by the DNA from domestic animals both from the laboratory and reagents[59]. To take this issue into account, we run a large number of controls (9 controls, with 8 PCR replicates each). The amplification of domestic animals in controls was extremely low (Supplementary Table 5 below). Amplification rate in controls was zero for most of mammals (*Bos*, *Sus scrofa*, *Equus*, *Cervus*), was low for *Ovis aries* (1.4% of controls), while it was moderate for *Capra hircus* (6.9% of controls). In order to take into account the risk of false positives, we run species detection occupancy modelling (SODM) that integrate the occurrence of both false-positive and false-negative errors (see Chen and Ficetola[15,60] and others[61]). These models estimate species occupancy and also the rate of false detections; the frequency of contamination into the controls (upper 99% credible interval) was included in SODM as prior for the frequency of false positives. The estimates of species occupancy reported in our manuscript are corrected taking into account the rate of false positives. In practice, each sample was analyzed in multiple PCR replicates, and was considered only if multiple PCR replicates confirm the sedaDNA detection.

**Species**	**Observed rate of contamination**	**95% CI**
Bos	0.00	0.00 – 0.03
Sus scrofa	0.00	0.00 – 0.03
Equus	0.00	0.00 – 0.03
Cervus	0.00	0.00 – 0.03
Ovis aries	0.013	0.001 – 0.06
Capra hircus	0.069	0.027 – 0.15

Table 5. Mammal results from negative controls given as the rate of contamination for the 6 detected species, as well as the estimated 95% CIs of the contamination rate (calculated as confidence intervals for binomial proportions using the Jeffreys' method[62]).

Additional References

53. Gigout Covex, C. et al. New insights on lake sediment DNA from the catchment: importance of taphonomic and analytical issues on the record quality. *Scientific Reports* 9, 14676 (2019).

54. Capo, E. et al. Optimizing the experimental procedure to analyze ancient DNA preserved within lake sedimentary archives. Guidelines for future research using lake sedimentary DNA to track historical changes in terrestrial and aquatic ecosystems. *Quaternary MDPI* (2021).

55. Haile, J., et al. (2007). "Ancient DNA chronology within sediment deposits: are paleobiological reconstructions possible and is DNA leaching a factor?" Molecular Biology and Evolution 24: 982-989.

56. Parducci, L., et al. (2017). "Transley Reviews: Ancient plant DNA from lake sediments." New Phytologist 214(3): 924-942.

57. Sjögren, P. *et al*. Lake sedimentary DNA accurately records 20th Century introductions of exotic conifers in Scotland. *New Phytologist* 213, 929-941 (2017) doi:10.1111/nph.14199

58. Birks, H.H. & Birks, H.J.B. Multi-proxy studies in palaeolimnology. *Vegetation History and Archaeobotany* 15: 235-251 (2006) 10.1007/s00334-006-0066-6

59. Leonard, J. A., Shanks, O., Hofreiter, M., Kreux, E., Hodges, L., Ream, W., Wayne, R.K. & Fleischer, R. C. Animal DNA in PCR reagents plagues ancient DNA research. *Journal of Archaeological Science* 34, 1361-1366 (2007).

60. Chen, W. & Ficetola, F. Conditionally autoregressive models improve occupancy analyses of autocorrelated data : An example with environmental DNA. *Molecular Ecology Resources* 19, 163-175 (2019).

61. Lahoz-Monfort, J.J., Guillera-Arroita, G. and Tingley, R. Statistical approaches to account for false-positive errors in environmental DNA samples. *Molecular Ecology Resources* 16, 673–685 (2016).

62. Brown LD, Cai TT, DasGupta A. Interval estimation for a binomial proportion. *Stat Sci* **16**, 101-117 (2001).
